# Restoring rhythm to prevent age-related fractures

**DOI:** 10.18632/aging.204192

**Published:** 2022-07-19

**Authors:** Annelies E. Smit, Maaike Schilperoort, Elizabeth M. Winter

**Affiliations:** 1 Department of Medicine, Division of Endocrinology, Leiden University Medical Center, Leiden, The Netherlands; 2 Department of Medicine, Columbia University Irving Medical Center, New York, NY 10032, USA

**Keywords:** circadian rhythm, fractures, osteoporosis, glucocorticoids, chronotherapy

We all are aware of the physical decline that accompanies aging. A typical example is age-related bone loss, or osteoporosis, which markedly increases lifetime fracture risk and has a multifactorial origin, to which determinants as gender, diseases such as rheumatoid arthritis, and therapeutic glucocorticoid (GC) use all contribute. Osteoporotic fractures usually require long-term rehabilitation and care, and result in a significantly impaired quality of life with increased mortality. With the current trend of population aging, the societal burden and costs of osteoporosis have become a worldwide concern.

Among the many risk factors for osteoporosis, a new kid on the block is disruption of the biological clock. The biological clock dictates circadian (i.e., 24 hour) rhythms in metabolic organs including the skeleton, as reflected by oscillations in circulating bone turnover markers and gene expression patterns in bone [1]. Disruption of circadian rhythm by night shift work or irregular sleeping patterns has been associated with low bone mineral density (BMD) and increased fracture risk [2]. Importantly, we could confirm these findings in a preclinical model of circadian disruption by shifting light-dark cycles [1], establishing causality between rhythm disturbances and osteoporosis. After having established causality, it was essential to elucidate the mechanisms that underlie circadian bone remodelling. It is well-known that the endogenous GC hormone cortisol plays an important role in synchronizing rhythm in peripheral tissues, and a pioneering study showed that rhythmic gene expression patterns in bone are dampened in absence of GC signalling [3]. Circulating cortisol levels display a strong rhythm, and we recently demonstrated that flattening of this endogenous GC rhythm while remaining physiological cumulative levels results in osteoporosis [4], indicating that circadian variation in GC levels is essential for bone health.

Rhythm in circulating cortisol levels is regulated by the “master clock”, the suprachiasmatic nucleus (SCN) in the hypothalamus. SCN-driven signalling through the hypothalamus-pituitary-adrenal axis (HPA axis) results in adrenal cortisol secretion. Circulating GCs in turn exert negative feedback on the hypothalamus and pituitary gland. With aging, SCN rhythmicity dampens and becomes decoupled from environmental rhythms [5]. This dysregulates rhythmic cortisol secretion, indicated by a dampened amplitude and an earlier morning peak [6]. In addition, mean cortisol levels increase with aging, as the negative feedback response of the HPA axis to GCs declines [6). Since both SCN [5] and cortisol [6] rhythm amplitude decline with age, and because we demonstrated in a preclinical model that flat endogenous GC levels result in osteoporosis, we argue that flattened circadian rhythmicity in the elderly population is causally related to the high incidence of osteoporosis at older age [4]. Consequently, maintaining circadian rhythmicity in elderly could be very important to prevent age-related osteoporotic fractures.

Lifestyle interventions that reinforce rhythmic output of the SCN may provide a cost-efficient and durable option to prevent age-related osteoporosis. The SCN receives input from internal and environmental “cues”, which together dictate SCN rhythmic output. The predominant regulator of SCN output is light, which signals directly to the SCN through light-sensitive photoreceptors. As older individuals experience reduced sensitivity to light, e.g., due to lens clouding and a reduced number of photoreceptors, SCN neuroendocrine response to light may dampen accordingly [5]. Light therapy, consisting of increased exposure to high intensity light during daytime, may thus reinforce synchronization of SCN rhythm to 24h light/dark cycle. Sleep/wake rhythm acts as another environmental SCN cue, which in its turn is regulated by SCN rhythm. Elderly commonly experience poor sleep quality [6], and sleep disturbances at elder age have been linked to an increased osteoporosis risk [7]. Thus, restoring normal sleep/wake cycles by psychological and behavioural measures, such as strict bedtime routines, may strengthen SCN rhythm. Furthermore, aside from the SCN, peripheral organs and brain areas act as circadian pacemakers that can be synchronized to external rhythms and to each other. Unlike light, eating and exercise patterns mainly regulate circadian rhythmicity directly in peripheral organs, including bone [8]. In elderly, physical inactivity and irregular eating patterns are common, and both have been demonstrated to dysregulate bone rhythm [8]. Regular eating and exercise patterns, tailored to a person’s physical capabilities, may reinforce peripheral circadian rhythmicity and protect bone health [2].

At current, calcium/vitamin-D supplementation and antiresorptive or anabolic medication form the cornerstone of osteoporosis treatment. Whereas lifestyle changes like timed exercise and timed feeding may contribute to reinforce circadian rhythmicity, we propose to incorporate chronotherapy, i.e., coordinating the time of drug intake to circadian rhythm, as this may optimize efficacy and tolerability [2]. This is for example demonstrated by the fact that morning administration of bone formation-promoting teriparatide further increased BMD, as compared to evening administration [11. Given the importance of GC rhythm to bone remodelling, treatment regimens that improve circadian GC rhythm may provide another option to prevent age-related osteoporosis, especially for patients receiving GC therapy [4].

**Figure 1:**
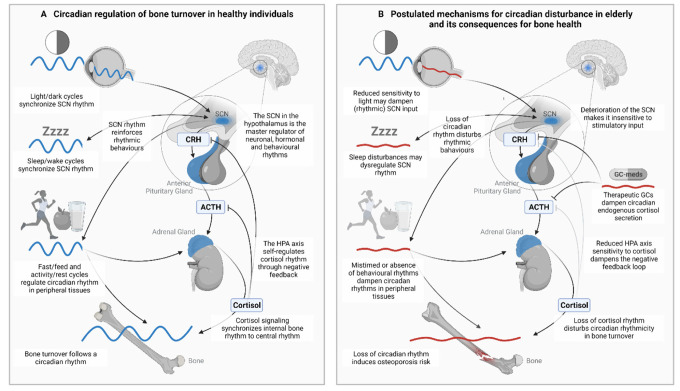
**Bone health depends on diurnal variation in bone turnover, which is regulated by the circadian timing system.** (**A**) With aging circadian rhythmicity is disturbed, which may contribute to osteoporosis (**B**). SCN= Suprachiasmatic nucleus, GC= glucocorticoid, HPA axis= hypothalamic-pituitary-adrenal axis. Figure created with BioRender.com

In conclusion, the multifaceted origin of age-related fractures asks for a full toolbox of intervention strategies, to which restoring circadian rhythm may provide a valuable addition. Lifestyle and medical interventions may improve sleep quality and decrease risk for osteoporotic fractures (Figure 1). Furthermore, respecting circadian timing through chronotherapy could optimize current and new therapeutic outcomes.
